# Putting a premium on altruism: A social discounting experiment with South African university students

**DOI:** 10.1371/journal.pone.0196175

**Published:** 2018-04-17

**Authors:** Frederik Booysen, Sevias Guvuriro, Alistair Munro, Tshepo Moloi, Celeste Campher

**Affiliations:** 1 Population Health, Health Systems and Innovation, Human Sciences Research Council, Pretoria, South Africa; 2 Department of Economics, University of the Free State, Bloemfontein, South Africa; 3 National Graduate Institute for Policy Studies, Tokyo, Japan; Universidad Loyola Andalucia, SPAIN

## Abstract

This paper reports on a social discounting experiment conducted with university students in South Africa. In line with other social discounting task experiments, participants identify target individuals at different degrees of intimacy in their social network and then make 10 choices involving sums of money for themselves or their targets. For an altruism premium to exist, senders’ donations to recipients should be positive, statistically and economically significant, and independent of relationship closeness. We hypothesize that in addition to the altruism premium for kin documented in the literature, there may be other premia for family in general and for partners and friends. We find that, apart from the “kinship” premium, there is a sizeable “intimacy” premium, which together translates into a substantial “family” premium. The study also finds a “friendship premium”, as is documented in various experiments. The closeness of relationships among family and kin, especially close kin, has a significant and large effect on altruism. The results also attest to the importance of the extended family in regards to the “kinship” premium on altruism. These various premiums on altruism emphasise the importance of the supportive role of various social systems. Nevertheless, altruism within families and among close kin might also be enhanced by building more cohesive and stronger families using developmental social welfare programmes.

## Introduction

Pro-sociality is an important feature of human behaviour [1–2]. There is substantial experimental evidence on the negative associations between altruism or giving, and social distance [3–10]. These experiments, which all employ the dictator game (DG) and/or ultimatum game (UG) to elicit some measure of altruism, operationalise social distance by either conducting social network analysis [5,7–8] or through providing certain social information regarding the recipients (second movers) to senders (first movers) [3–4,6,10], or both [9]. This identification strategy is either implemented as a randomised treatment(s) [4,6] or provided as contextual framing [3,5,7–10]. By design, though, the majority of these studies–six of the eight studies use student populations–only allows for an analysis of friendship networks [4–9]. The social discounting task, however, offers a more nuanced and diverse representation of social relations, extending beyond relationships within the subject pool. In fact, the negative association between altruism and social distance has also been firmly established in the early methodological work on social discounting [11–13] and the more recent applied literature in this field [14–16]. Yet, only a handful of social discounting experiments have documented the importance of kinship and family in inter-personal altruism [17–21].

There are two gaps in this literature. With the exception of the work by Strombach and her fellow researchers [21], none of the social discounting studies provide a more detailed analysis of how altruism is influenced by the nature and general quality of the relationship between sender and recipient [22]. Nor has the social discounting literature provided any test of the existence of a kinship “premium”, i.e. whether “individuals might be willing to act altruistically to kin to whom they are not close” emotionally [23]. In order to answer these questions it is necessary to collect information on the nature of the subject’s relationship with the recipient at each social distance. The experiment reported in this paper, unlike the majority of social discounting experiments, collected a variety of such detail when administering the social discounting task, using a short questionnaire. Relationship quality, therefore, which is not properly defined in this literature, here is represented by a more comprehensive measure, namely relationship closeness [24–27], extending beyond a single measure of emotional closeness, as relationship quality is defined in this literature [23]. The paper also makes contribution to this literature by investigating whether premiums of this nature extends beyond kin to spouses and partners, and to friends as well as to family in general. This paper therefore extends research on altruism and relationship closeness and on the kinship and other altruism premiums by using a social discounting task to measure altruism towards individuals at different points in the extended social network and by collecting detailed information on the nature of the relationship between the sender and each recipient. The research question, to summarise, is whether there is a premium on altruism expressed towards various social relations, i.e. whether subjects send significantly greater amounts to those closer to them even after accounting for relationship closeness. The experimental research is conducted in a laboratory setting with South African university students. The study, in addition to confirming the presence of a “kinship” premium, finds a sizeable “intimacy” premium, which together with the “kinship” premium translates into a substantial “family” premium. The study records a strong “friendship” premium on altruism. The study also confirms the importance to altruism of the closeness of relationships, especially among family and close kin.

## Materials and methods

### Participants

In this conventional laboratory experiment, the subjects are 113 under-graduate students at the University of the Free State in South Africa. There are approximately fifty percent more females than males in the sample: 59.2% versus 40.7%. Subjects’ mean and median ages are 22.5 and 22 years, respectively [IQR 21–24]. Four subjects only had previously participated in an experiment of this nature.

### Instrumentation

In accordance with the study protocol, a pencil-and-paper instrument including Rachlin and Jones’ standard social discounting task was administered to study participants [https://www.protocols.io/private/b8442b4ef81c976fd8496e398c8543dd] (S1 File) [13]. Social distance was operationalised by asking subjects to mentally envisage “a list of the 100 people closest to you in the world ranging from your dearest friend or relative at #1 to a mere acquaintance at #100”. Subjects then completed a series of seven uniform tables, one for each social distance (1, 2, 5, 10, 20, 50, and 100). In each table, subjects had to select either A or B in each of the ten rows, as illustrated in Table 1. The task was counter-balanced, with the tables in half of the experimental packages organised in the standard ascending order and in the other half in descending order in terms of the seven social distances. The maximum offer to subjects of R180 decreases in R20 increments as one moves up the table, whereas the offer to recipients remain constant at R160.

**Table 1:** Social discounting task–person #1.

Row	Option A:	Option B:	Choice	
1.	R180 for you alone	R160 for person #1 on the list	A	B
2.	R160 for you alone	R160 for person #1 on the list	A	B
3.	R140 for you alone	R160 for person #1 on the list	A	B
4.	R120 for you alone	R160 for person #1 on the list	A	B
5.	R100 for you alone	R160 for person #1 on the list	A	B
6.	R80 for you alone	R160 for person #1 on the list	A	B
7.	R60 for you alone	R160 for person #1 on the list	A	B
8.	R40 for you alone	R160 for person #1 on the list	A	B
9.	R20 for you alone	R160 for person #1 on the list	A	B
10.	R0 for you alone	R160 for person #1 on the list	A	B

At the general level, this task measures social behaviour, i.e. choices with fitness consequences for an actor (subject/sender) and another individual (recipient) [28]. Methodologically, the social discounting choice task speaks specifically to the notion of altruism, as defined by Hamilton [29] and Trivers [30], i.e. a behaviour that in the short term is potentially costly to the actor (i.e. foregoing the amount in option A) and potentially beneficial to the recipient (i.e. receiving the amount in option B), also described as a “-/+” action [28].

### Questionnaires

Subjects completed two short questionnaires, providing information on the person occupying each social distance [‘recipient characteristics’–S2 File] and personal socio-demographic and related information [‘sender characteristics’–S3 File].

### Ethics

Ethical clearance for the study was obtained from the Faculty of Humanities at the University of the Free State (UFS-HUM-2015-74). Participation was voluntary and written informed consent was obtained from all subjects.

### Payment

Subjects received a show-up fee of R30 (Session 1) or R50 (Session 2). Show-up fees were increased between sessions to facilitate greater participation. On completion of the experiment, a within-subject random incentive system (RIS) was used to calculate each subject’s individual earnings [31]. First, one of the seven social distance tables was selected randomly. In the next step, one of the ten rows in this specific table was selected randomly. Finally, the subject’s actual choice on this selected row was implemented. Where option A was selected, the relevant amount is paid to the subject only, as per the choice task (Table 1). Where option B was selected, only the nominated recipient at the relevant social distance is subsequently paid the relevant amount. Each subject met individually with the experimenter or assistant to implement the randomised payment process. Subjects on average earned an additional R150. Payments were made in private and via mobile phone, for cash withdrawal at an ATM.

### Data

The complete dataset is available at: http://dx.doi.org/10.17632/k2pj67kvzf.2. The full survey includes data for a total of 791 tasks completed by a total of 113 subjects (seven tasks per subject). Subject-level information includes the basic socio-demographic and other characteristics of study participants collected with the aid of the subject questionnaire (S3 File). The task-level data captures the crossover values for each table as well as the details collected on each recipient (S2 File).

### Measures

The social discounting task is “a powerful means of quantifying altruism in humans” [32]. *Altruism*, the dependent variable, is measured as, “the amount of money a participant [is] willing to forego to give a fixed amount of money to another person” situated at a specific social distance [13]. The corresponding crossover value, the primary dependent variable in this study, is the specific point at which the subject first chooses B on each table. For example, if a subject chose the selfish option at R180 for me or R160 for you (row 1) and switched to the generous option at R160 for me or R160 for you (row 2), the crossover point is calculated as R160. If the subject switched between R40 for me or R160 for you (row 8) and R20 for me or R160 for you (row 9), the crossover point is R20. In case option B was selected throughout, the crossover point is assumed to be R180. Where option A was selected throughout, the crossover point is assumed to be zero (i.e. no altruism).

Inconsistent preferences remain a limitation with MPL-type elicitation tools such as the social discounting task. Among the 791 tasks completed by the 113 subjects, 149 tables or 18.8% of tasks saw the subject switch multiple times from A to B, which may be indicative of a lack of understanding. Such errors, in terms of bias, would see the extent of altruism overestimated, as is the case here. The mean crossover value for tasks with multiple switches is R153, compared to R87 for tasks without multiple switches (t = 14.06, p<0.001). One also would not expect subjects to select A throughout, because the last option in row 10 is a choice between zero for oneself and R160 for the other person. Altruism should prevail and subjects preferring A over B in row 1–9 should be switching to B in the final row. Yet, envious or spiteful subjects may choose to withhold R160 from another person, especially those at further social distances. Alternatively, subjects may not have fully understood the task. In this study, A was selected throughout in only 2.8% of tasks, i.e. 22 out of 791 tasks. In this case, however, the measure of altruism would err on the conservative side and represent an underestimate of giving. Given this inconsistency in preferences, the descriptive and regression analysis outlined below is only applied to the sub-sample of tasks with consistent responses. The analytical sample includes the tasks exhibiting no switches, because such responses may be consistent with the complete absence of altruism. Lending credibility to this claim is the fact that almost three quarters of these responses (n = 16) pertain to tasks for distances 50 and 100. The analytical sample, therefore, comprises a total of 642 choice tasks completed by 107 subjects (6 subjects switched multiple times in all seven the tasks).

Three categorical variables that are characteristic of social and family relations are used in the analysis. Based on the questionnaire’s response options, the variable *relationship* distinguishes between spouses and partners, parents, siblings, other family, friends, neighbours and acquaintances, strangers (someone not known to the sender by name), and others. *Relation*, the second variable, constructed from the variable relationship and employed in the main hypothesis tests, distinguishes five types of sender-recipient relationships. These are ‘kin’ (parents, siblings and other family), ‘partners’ (spouses and partners), ‘friends’, and ‘others’ (neighbours, acquaintances, strangers and others). ‘Family’ represents the combination of ‘kin’ and ‘partners’. The third classification is based on three categories of the so-called coefficient of genetic relatedness (*r*). The categorical variable *relatedness*, applied to kin, takes on a value of ‘1’ for other genetic relations (r = 0.125), ‘2’ for uncles/aunts, nephews/nieces, and grandparents (r = 0.25), and ‘3’ for parents and siblings (r = 0.5) [20].

*Relationship closeness*, an independent variable represented by a normalised composite index constructed with the aid of Multiple Correspondence Analysis (MCA), is used to proxy the nature and quality of relationships between senders and their recipients. The index is derived from (a) a 10-point scale on emotional and psychological closeness, and three other variables, namely (b) how long the sender has known the recipient, (c) the frequency of communication between sender and recipient, and (d) the physical proximity of sender and recipient in terms of current living arrangements (S2 File). Methodologically, the index is linked to Kelley’s interdependence model of relationship closeness, which postulates that ‘interdependence’ is characteristic of close relationships and that ‘closeness’ has four dimensions, namely strength, frequency, diversity, and duration or longevity [24–27]. Thus, ‘interdependence’ can be hypothesised to increase when emotional and psychological ties between sender and recipient strengthen (strength), when the frequency of their communication increases (frequency), when the sender and recipient have known each other for longer (duration/longevity), and when the sender and recipient are co-resident (which may facilitate diversity).

### Hypotheses

The study tests four primary hypotheses. First, in accordance with the work of Curry, Roberts and Dunbar [23], the analysis tests for the presence of a “kinship premium”, i.e. whether subjects are more altruistic to kin, independent of relationship closeness (*hypothesis 1*). The second and third hypotheses seeks an answer to the question as to whether there are non-kinship premiums on altruism, i.e. for spouses and partners (“intimacy” premium) (*hypothesis 2*) and for friends (“friendship” premium) (*hypothesis 3*). The paper also tests for a “family” premium (*hypothesis 4*), where kin and partners are jointly classified as family. The paper, therefore, tests for the presence of four premiums, namely a “kinship”, “intimacy”, “friendship” and “family” premium. Furthermore, following kin selection theory, the “kinship premium” is also assessed in terms of genetic relatedness, first in comparison to ‘others’ and then between the different coefficients of relatedness (*r*), which provides an inter-generational context to the analysis. A secondary and overarching hypothesis is that inter-personal altruism will increase with greater relationship closeness within each comparative social group (*hypothesis 5*), e.g. that altruism towards kin will increase with greater relationship closeness among kin.

### Analysis

The statistical analysis investigating the various hypotheses comprises two components. The first is a descriptive component and the second multivariate regression analysis. As a precursor to the regression analysis, the main dependent variable (crossover value, Rand) is compared across social and family relationships, using t-tests and F-tests. To assess the robustness of the findings, the regression analysis employs two techniques, the first being standard ordinary least squares (OLS) regression. As crossover values are not distributed normally (Shapiro-Wilk test: W = 0.9903, p<0.001; Shapiro-Francia test: W´ = 0.9908, p<0.001), the study also employs quantile regression analysis, which is more suitable for the analysis of data that do not fit a normal distribution. Three quantile regression models are estimated, one at the median (q = 0.50) and one each at the two quartiles (q = 0.25 and q = 0.75).

Four sets of multivariate regression models are employed to test the various hypotheses. First, four regression models are estimated to draw the following comparisons, namely family versus others, kin versus others, partners versus others, and friends versus others, where “others” represent neighbours, acquaintances, strangers and recipients classified as ‘other’. In order to further verify the existence of the kinship, intimacy and family premiums on altruism, a further four comparisons are drawn, namely between family versus friends, kin versus friends, kin versus partners, and partners versus friends. Next, three more regression models are estimated to test for kinship premiums based on the coefficients of relatedness (*r*) and in comparison to “others”. Lastly, three more regression models are estimated to test for altruism premiums among kin. Here, a comparison is drawn between the three coefficients of relatedness (*r*), i.e. 0.5 versus 0.25, 0.5 versus 0.25, and 0.25 versus 0.125. In each case, the regression model includes a corresponding dummy variable reflecting the relevant binary comparison. To determine the association between altruism and the closeness of relationships within the target group (*hypothesis 5*), the relevant relationship dummy variable is interacted with the relationship closeness scale.

The regression models control for selected sender and recipient characteristics. Sender characteristics include age, gender, household poverty, and previous participation in experiments. Recipient characteristics include age and gender. For the kinship-based analysis across genetic relatedness, four additional subject-level controls are included, namely FACESIV’s cohesion and flexibility ratio scales of family functioning [33] and the Family Communication (FCS) and Family Satisfaction (FSS) scales [34].

The experiment included a payment “treatment”, the goal of which was to assess if incentivising the social discounting task (SDT) impact on measures of altruism. While there is evidence that payment introduced some bias, running the analysis reported on here only with the sub-sample of paid or unpaid subjects effectively would half the sample size, thus precluding many of the comparative sub-group analysis. For this reason, this feature of the experiment is also adjusted or controlled for in the regression analysis rather than applying the analysis to a smaller sub-sample of subjects. The analysis also controls for ‘Session’ insofar as the experiment was conducted in two separate sessions taking place on different days. At the same time, controlling for session takes into account the differences in show-up fees, which may impact incentive structures and hence influence not only who participates in the experiment, but also subjects’ decisions in the experimental tasks. Furthermore, because inconsistent preferences are relatively high at subject level (48.6% of subjects switched multiple times or not at all on at least one of the seven tasks) and may signify a general lack of understanding, the regression analysis also controls for subject-level inconsistency in preferences, drawing a distinction between multiple and zero switches.

## Results

**Fig 1:**
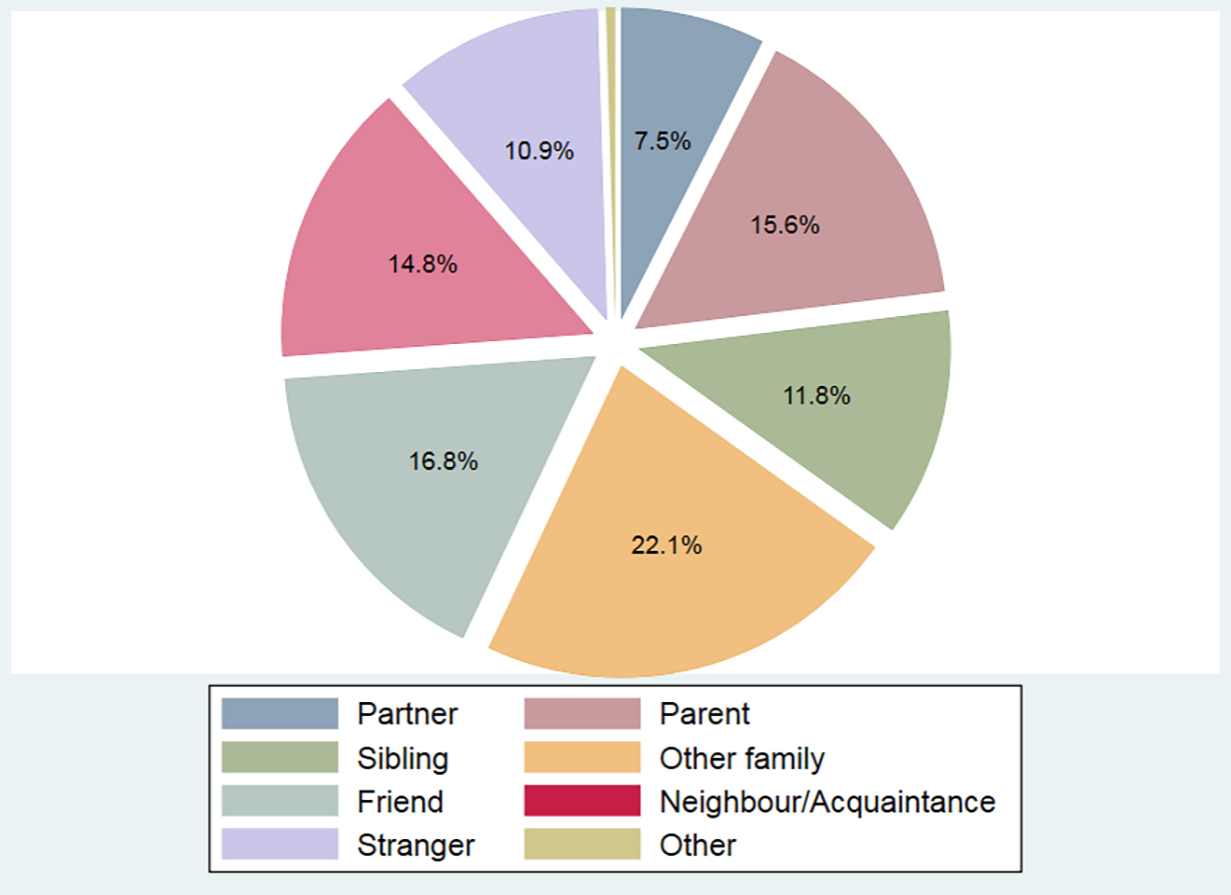
Subject’s relationship to recipient.

Fig 1 presents the composition of the recipients identified by subjects in terms of relationship. Half of the recipients represent kin of the sender. Almost a third of kin are parents (31.4%) and almost a quarter are siblings (23.9%). Another quarter (25.2%) are uncles/aunts or nephews/nieces, with a small share of grandparents (6.3%). Given that subjects are full-time under-graduate students and therefore relatively young (mean age = 22 years), relatively few recipients are husbands/wives or boyfriends/girlfriends to the subject (7.5%). Among the remaining recipients, 39.1% are friends, 34.4% are neighbours or acquaintances, and 25.4% are anonymous strangers (i.e. someone not known to the sender by name).

**Fig 2:**
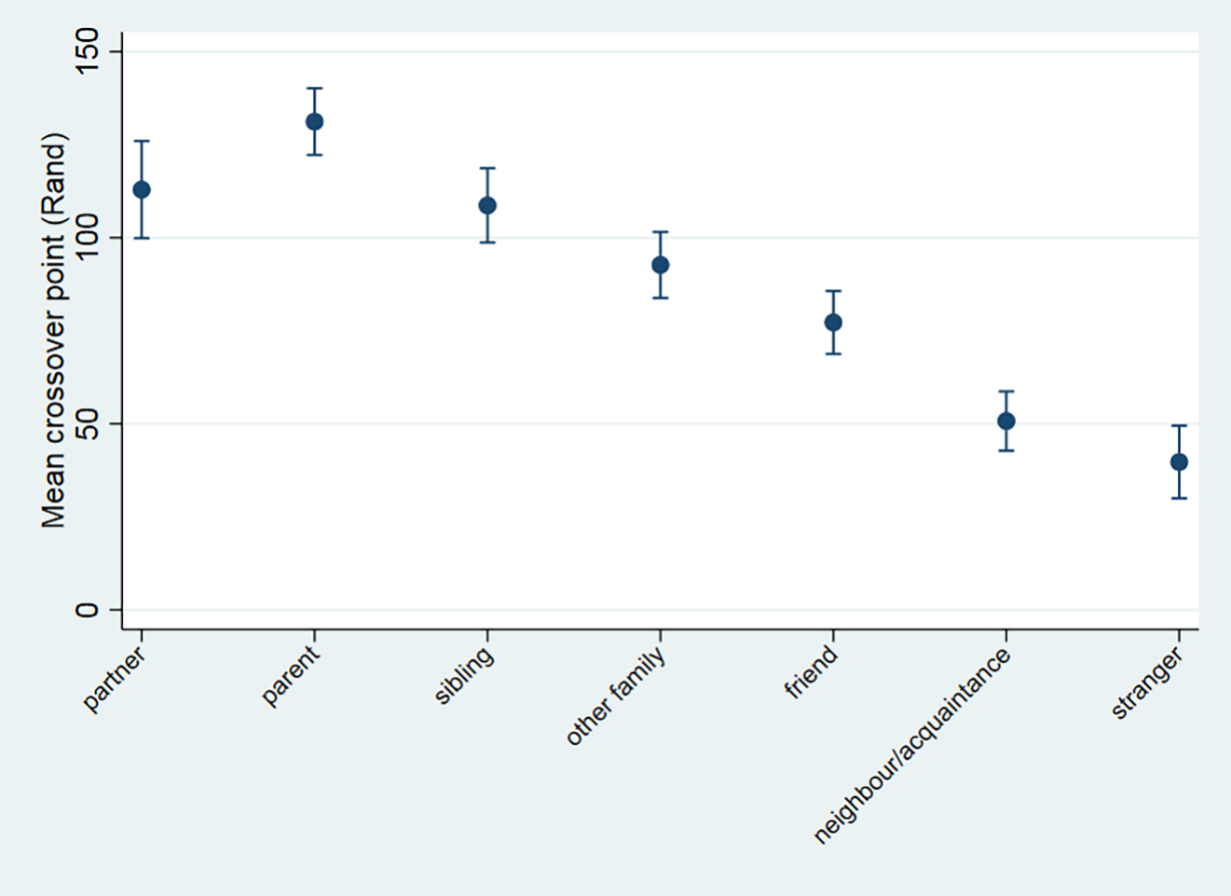
Crossover value (Rand), by relationship.

**Fig 3:**
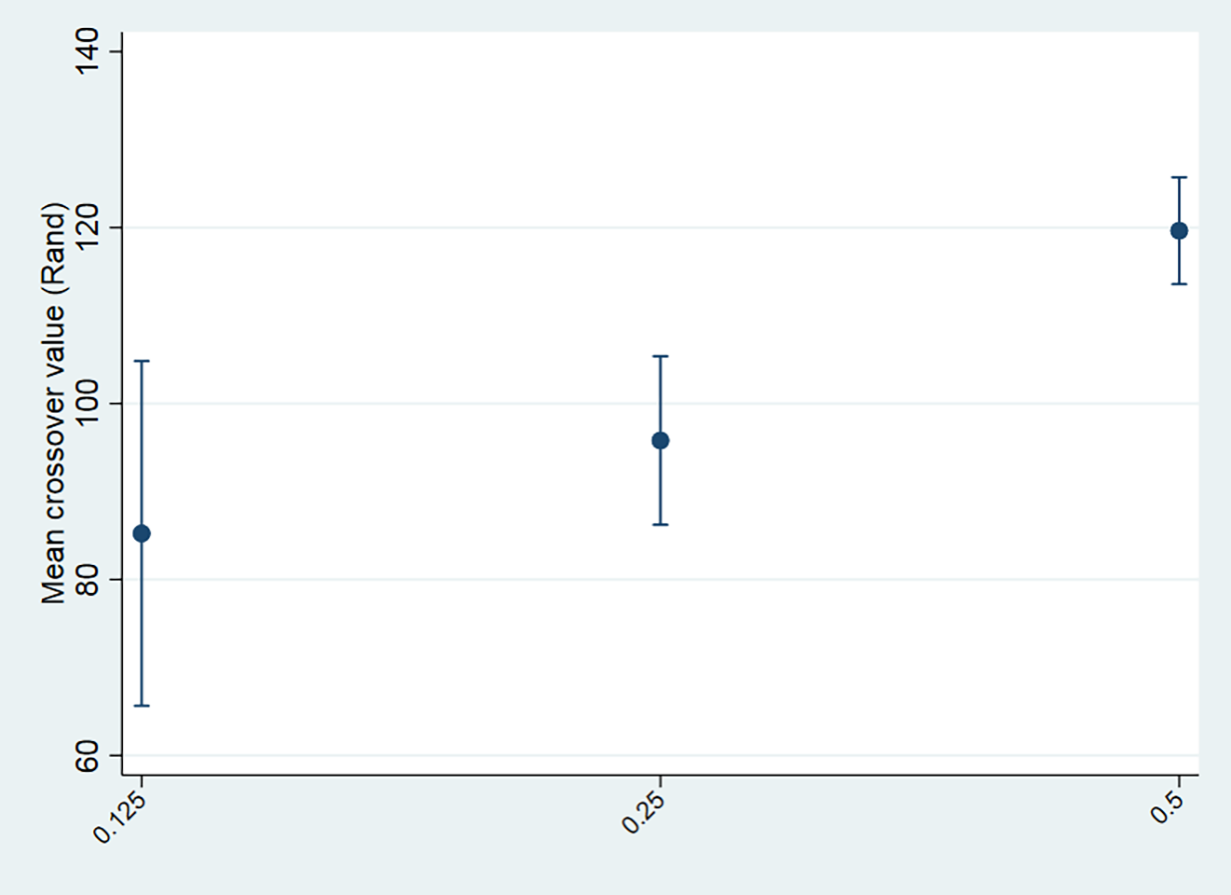
Crossover value (Rand), by relatedness.

The graphical representation of crossover values in relation to relationship and relatedness exhibit the expected associations, i.e. altruism falls as relationships become more socially distant (Fig 2) (F = 43.66, p<0.001), while altruism increases as relatedness rise (Fig 3) (F = 13.59, p<0.001). Whereas there is no significant difference in altruism between more distant kin (*r* 0.25 versus *r* 0.125: t = 1.06, p = 0.144), altruism toward close kin is significantly greater than toward more distantly related kin (*r* 0.5 versus *r* 0.25: t = 4.20, p<0.001; *r* 0.5 versus *r* 0.125: t = 4.11, p<0.001).

The ordinary least squares (OLS) regression results reported in Table 2 bring to light two important observations. The first main result is that relationship closeness among family and kin has a significant and large effect on altruism (*hypothesis 5*). For family and kin, a one standard deviation increase in relationship closeness increases the crossover value by as much as R22. Relationship closeness among partners and friends has no bearing on reported inter-personal altruism. The second and perhaps most important finding is that there are sizeable premiums on altruism. When compared to ‘others’, the “intimacy” premium is the largest (R45) (*hypothesis 2*), followed by the premiums on family (R44) (*hypothesis 4*), kinship (R42) (*hypothesis 1*), and friendship (R14) (*hypothesis 3*), respectively. There are no significant premiums for kin over partners when using OLS regression models to estimate the premium. (The latter results are not reported here due to constraints of space, but are available from the corresponding author on request.)

**Table 2:** Altruism between family, kin, partners, friends and others.

	OLS regression	Quantile regression (q)		
		0.25	0.50	0.75
Family *vs* Others	44.231** (7.434)	17.091 (9.876)	44.517** (15.516)	58.601** (9.753)
Family * Closeness	22.862* (9.426)	64.088** (19.225)	26.645 (29.180)	23.446 (12.039)
F-statistic (p-value)	26.66 (<0.001)			
(Pseudo) R^2^	0.371	0.233	0.232	0.258
Sample (n)	534	534	534	534
Kin *vs* Others	42.277** (7.611)	16.552 (10.969)	42.648** (15.938)	59.551** (9.888)
Kin * Closeness	22.978* (9.479)	56.168** (22.314)	27.489 (29.795)	23.574* (11.508)
F-statistic (p-value)	25.65 (<0.001)			
(Pseudo) R^2^	0.374	0.228	0.232	0.274
Sample (n)	486	486	486	486
Partners *vs* Others	45.557* (18.652)	22.591 (35.699)	65.930 (33.368)	77.833** (25.938)
Partner * Closeness	27.434 (35.929)	78.676 (67.261)	-1.257 (64.145)	-18.936 (48.829)
F-statistic (p-value)	18.44 (<0.001)			
(Pseudo) R^2^	0.447	0.264	0.307	0.354
Sample (n)	216	216	216	216
Friends *vs* Others	14.521* (5.883)	15.652 (8.451)	17.847** (6.633)	12.970 (10.887)
Friend * Closeness	14.715 (8.457)	8.205 (17.920)	12.308 (10.689)	24.218* (10.098)
F-statistic (p-value)	14.72 (<0.001)			
(Pseudo) R^2^	0.347	0.200	0.216	0.253
Sample (n)	276	276	276	276

The dependent variable is the crossover value (Rand). Robust standard errors are reported in parentheses. “Family” includes partners and spouses together with all kin. “Others” includes neighbours, acquaintances, strangers and recipients classified as ‘other’. Statistical significance

** 1%

* 5%.

The quantile regression results for the most part mirror these findings, though there are interesting differences in terms of how each of the associations vary across the distribution of crossover values. The kinship (*hypothesis 1*) and family (*hypothesis 4*) premiums are statistically significant, not only at the median (q = 0.50), but also among the more altruistically inclined (q = 0.75), where the two premiums are approximately R14 and R17 greater in magnitude, respectively. The intimacy premium (*hypothesis 2*) is only significant for the more altruistic subjects (q = 0.75), but also substantially larger in size (R78). The friendship premium (*hypothesis 3*), at R18, in turn is significant only at the median quartile. The friendship premium, as expected, is substantially smaller than the kinship and family premiums. The quantile regression results in Table 2 also brings to light one additional finding, namely that altruism towards friends, increases as relationship closeness increase (*hypothesis 5*). Where the closeness index increases by one standard deviation, giving to friends increases by R24. The relationship closeness effect for kin and family (*hypothesis 5*) is present among those subjects who are less altruistic (q = 0.25) and then more than twice the size in magnitude (>R56). In addition, altruism towards kin also increases with relationship closeness among the more altruistic, at R23 per one standard deviation increase in the index of relationship closeness (q = 0.75).

According to the OLS regression results presented in Table 3, the kinship (*hypothesis 1*) and family (*hypothesis 4*) premiums retain their statistical significance when the comparison group is friends rather than others. Based on the results of the corresponding quantile regression analysis, greater premiums are observed only among more altruistically inclined subjects (q = 0.75). This is the case for all three premiums, i.e. kin (hypothesis 1), partners (hypothesis 2), and family (hypothesis 4). The intimacy premium (R74) by far outstrips the kinship (R30) and family (R33) premiums. Interestingly, the closeness of relationships among kin, family and partners do not have any significant impact on giving in these comparisons with friends (*hypothesis 5*).

**Table 3:** Altruism between family, kin, partners and friends.

	OLS regression	Quantile regression (q)		
		0.25	0.50	0.75
Family *vs* Friends	20.975** (7.110)	1.711 (12.236)	21.649 (16.146)	33.738** (10.831)
Family * Closeness	13.208 (13.480)	47.906 (29.563)	22.116 (32.075)	-6.709 (19.138)
F-statistic (p-value)	10.31 (<0.001)			
(Pseudo) R^2^	0.215	0.134	0.123	0.142
Sample (n)	474	474	474	474
Kin *vs* Friends	19.001* (7.290)	-2.149 (13.600)	14.642 (16.396)	30.177** (11.316)
Kin * Closeness	14.015 (13.334)	48.474 (30.926)	28.598 (32.928)	-5.973 (19.130)
F-statistic (p-value)	10.45 (<0.001)			
(Pseudo) R^2^	0.222	0.131	0.132	0.157
Sample (n)	426	426	426	426
Partners *vs* Friends	32.164 (19.624)	2.042 (42.457)	48.882 (36.777)	74.227** (24.983)
Partner * Closeness	8.582 (40.962)	48.692 (86.366)	-29.745 (70.843)	-46.486 (50.497)
F-statistic (p-value)	10.75 (<0.001)			
(Pseudo) R^2^	0.400	0.202	0.253	0.326
Sample (n)	156	156	156	156

The dependent variable is the crossover value (Rand). Robust standard errors are reported in parentheses. “Family” includes partners and spouses together with all kin. Statistical significance

** 1%

* 5%.

The attention now shifts to Table 4, where each level of genetic relatedness (*r* = 0.5, 0.25, 0.125) is compared to “others”. For starters, these results confirm hypothesis 5. Relationship closeness is important among close kin (r = 0.5), but mainly among subjects with moderate to low levels of altruism (q≤0.50). The OLS regression analysis shows significant kinship premiums at the two lower levels of genetic relatedness. More specifically, the crossover point is approximately R37 greater when comparing giving to uncles/aunts, nephews/nieces and grandparents to giving to others. For other genetic relations, the premium takes on a value of R32. These premiums occur particularly among the more altruistically inclined. At the upper quartile (q = 0.75), the magnitude of the kinship premiums are R54 (r = 0.25), and R70 (r = 0.125), respectively. Interestingly, therefore, kinship premiums are higher at greater levels of genetic relatedness, hinting at the relative importance of the extended family.

**Table 4:** Altruism between kin and others.

	OLS regression	Quantile regression (q)		
		0.25	0.50	0.75
r = 0.50 *vs* Others	25.531 (15.228)	4.630 (21.748)	14.339 (19.880)	39.529 (20.896)
r = 0.50 * Closeness	72.970** (27.068)	102.373* (41.484)	104.769** (34.778)	63.268 (35.921)
F-statistic (p-value)	28.31 (<0.001)			
(Pseudo) R^2^	0.513	0.315	0.356	0.351
Sample (n)	392	392	392	392
r = 0.25 *vs* Others	36.743** (7.047)	20.252 (16.991)	19.451 (17.713)	54.971** (20.148)
r = 0.25 * Closeness	6.718 (15.548)	41.226 (45.328)	21.802 (42.857)	11.656 (40.045)
F-statistic (p-value)	16.31 (<0.001)			
(Pseudo) R^2^	0.349	0.231	0.207	0.255
Sample (n)	268	268	268	268
r = 0.125 *vs* Others	31.586** (11.591)	4.995 (12.510)	17.178 (23.097)	70.032** (25.747)
r = 0.125 * Closeness	1.862 (9.509)	-4.810 (21.245)	7.833 (46.731)	-50.597 (56.740)
F-statistic (p-value)	7.64 (<0.001)			
(Pseudo) R^2^	0.275	0.143	0.175	0.215
Sample (n)	210	210	210	210

The dependent variable is the crossover value (Rand). Robust standard errors are reported in parentheses. “r” is the coefficient of relatedness. “Others” includes neighbours, acquaintances, strangers and recipients classified as ‘other’. Statistical significance

** 1%

* 5%.

The analysis of altruism among kin, i.e. comparisons based on genetic relatedness (Table 5), yields only one important result. In the OLS regressions, inter-generational relationship closeness only matters among close kin, i.e. children and parents and siblings (r = 0.5), with giving increasing as closeness increases. The quantile regressions in turn show only a significant result for the comparison with more distant genetic relations (r = 0125), including among less altruistically inclined subjects. No kinship premiums are observed when focusing on comparisons between kin, i.e. giving is not independent of the closeness of relationships.

**Table 5:** Altruism among kin.

	OLS regression	Quantile regression (q)		
		0.25	0.50	0.75
r = 0.50 *vs* r = 0.25	-8.483 (14.962)	-25.425 (29.831)	-17.045 (29.560)	-13.471 (28.018)
r = 0.50 * Closeness	61.649* (29.104)	96.697 (67.851)	86.840 (61.002)	48.483 (53.442)
F-statistic (p-value)	5.49 (<0.001)			
(Pseudo) R^2^	0.213	0.128	0.146	0.129
Sample (n)	324	324	324	324
r = 0.50 *vs* r = 0.125	-27.840 (20.503)	-19.823 (28.493)	-23.909 (31.861)	-20.892 (30.446)
r = 0.50 * Closeness	113.208** (34.640)	148.086* (57.487)	146.815* (58.849)	96.863 (51.028)
F-statistic (p-value)	5.54 (<0.001)			
(Pseudo) R^2^	0.302	0.224	0.230	0.184
Sample (n)	218	218	218	218
r = 0.25 *vs* r = 0.125	11.508 (10.091)	3.824 (23.256)	19.476 (24.492)	-1.603 (20.713)
r = 0.25 * Closeness	-1.671 (20.741)	69.312 (60.926)	-17.905 (50.949)	-33.654 (41.918)
F-statistic (p-value)	5.12 (<0.001)			
(Pseudo) R^2^	0.238	0.155	0.198	0.273
Sample (n)	142	142	142	142

The dependent variable is the crossover value (Rand). Robust standard errors are reported in parentheses. “r” is the coefficient of relatedness. Statistical significance

** 1%

* 5%.

## Discussion

There is a clear pattern in terms of how altruism varies across social and family relationships, including genetic relatedness. Rachlin and Jones, for example, in terms of relatedness, also found that the amount foregone to grant another a specified monetary gift falls as the coefficient of relatedness (*r*) declines [20]. Boyer, Lienard and Xu in turn report a similar social discounting gradient across family, friends, acquaintances, and strangers in a series of cross-cultural laboratory and field experiments conducted in China, Kenya and the USA [17].

The inclusion in this analysis of family in general and of spouses and partners, rather than friends only, adds an extra dimension to the study. Apart from the “kinship” premium, there is a sizeable “intimacy” premium. The study also finds a “friendship premium”, as is documented in various experiments [4,9,35–36], while the “family” premium is considerably large. In terms of Curry, Roberts and Dunbar’s “kinship premium” [23], the results attest to the importance of kin and in particular the extended family [37]. The “kinship” premium is greater among more distantly related kin. Among close kin, inter-generational relationship closeness is a main driver of altruistic behaviour, especially among the less altruistically oriented.

The study has various limitations. Payment, in theory, should be anonymous where the recipient is paid, but the sender may disclose his or her identity to the person or the recipient in turn may ask the sender regarding the payment. Subjects were not asked or required to refrain from disclosing their identity to paid recipients. This lack of anonymity, or the process of identification, which has been shown to significantly impact donations in dictator games [38], translates into some loss of control, but is unavoidable given the nature of the task and the practicalities necessary to implement payment. The study therefore cannot at a methodological level identify the specific motives for subjects’ altruistic behaviour. For example, it is not possible to know if the observed giving is a result of pure, unconditional or reciprocal altruism, nor for that matter to determine whether allocations are due to enforced reciprocity, signalling or preference-based reciprocity [9] or to mutualism [28]. Nor can the design of this experiment, like an experiment by Ale, Brown and Sullivan [39], test between kin selection and reciprocal altruism. Another limitation is that the ‘closeness’ index is an imperfect proxy rather than a more reliable and valid measure of relationship closeness such as the Relationship Closeness Inventory (RCI) [25], the ‘Inclusion of the Other in the Self’ (IOS) experimental task [26], or the Unidimensional Relationship Closeness Scale (URCS) [40]. Another caveat of this study is the inconsistency in subjects’ response patterns in the social discounting task, which somewhat constrained the statistical power of the regression analysis. An important recommendation, therefore, is that the original social discounting choice task be modified so as to guide respondents to record only a single switching point (unless not switching at all) rather than recording an answer on every row of the table.

There are also other important avenues for further research. Chief amongst these, in response to the limitations of the artificiality of the laboratory setting, is the replication of experiments of this nature with larger samples and in field settings in countries with a variety of cultural environments. At a theoretical level, custom-made technical designs may aid in addressing the dilemma of attributing these premiums to specific genetic, psychological, sociological or biosocial theories or processes. Nevertheless, the observed behaviour can be ascribed to a small sample of young adults, who in their own right represents an important group in the societal and family hierarchy.

## Conclusion

The presence, not only of “family” and “kinship”-based premiums, but premiums for partners and friends, augurs well for the supportive role of various social systems and the safety nets they provide. Such altruism is necessary to avert and positively respond to the many relational and societal struggles and challenges of everyday life. The “kinship” premium moreover is common even in the extended family, emphasising the relevance and importance of this social institution in countries such as South Africa. Altruism among family and kin, within the context of inter-generational relationship closeness, can be enhanced by building more cohesive and stronger families, thus suggesting a role for developmental social welfare programmes such as marriage and relationship education. Further studies in this area should however take cognisance of the caveats of inconsistent responses, lack of anonymity and variation in show-up fees characteristic of the present study and through design implement appropriate strategies to address these limitations.

## Supporting information

S1 File Social discounting task. (DOCX)

S2 File Recipient questionnaire. (DOCX)

S3 File Subject questionnaire. (DOCX)
