# Supplementary figures and images for: Putting a premium on altruism: A social discounting experiment with South African university students
Source: PLoS One. 2018 Apr 17;13(4):e0196175. doi: 10.1371/journal.pone.0196175 (PMC5903621; doi:10.1371/journal.pone.0196175)

**Supplementary material S1:** Social Discounting Task (SDT) – standard table


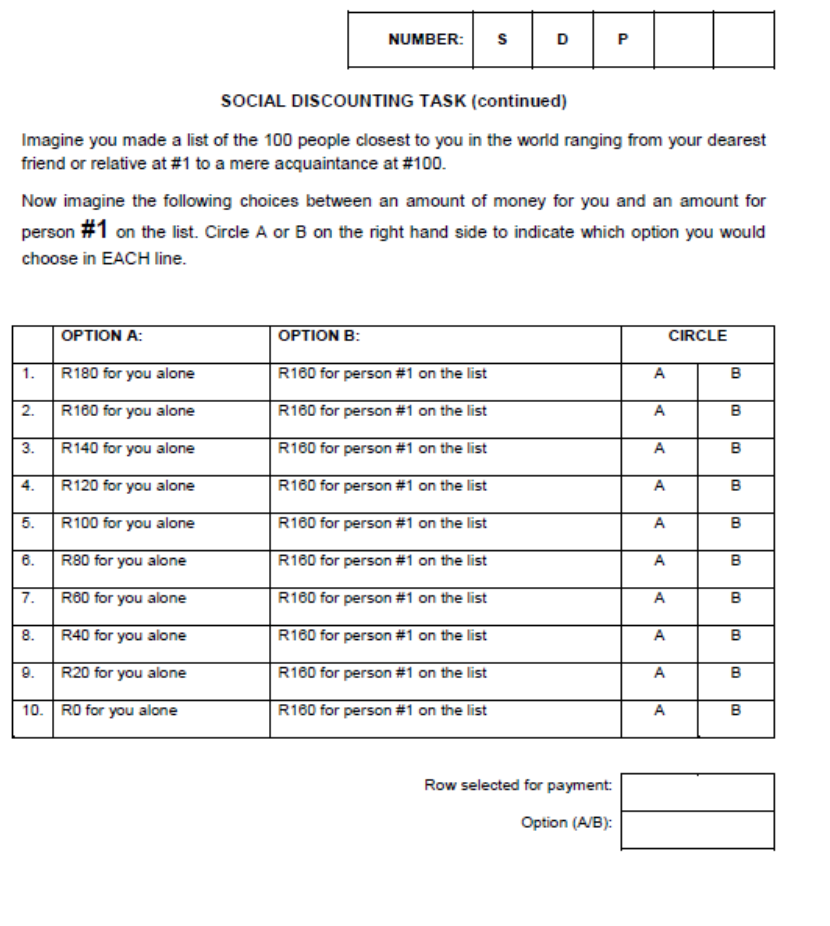

Supplement: S1 File — (DOCX) [file pone.0196175.s001.docx]
