# Supplementary material for: Putting a premium on altruism: A social discounting experiment with South African university students
Source: PLoS One. 2018 Apr 17;13(4):e0196175. doi: 10.1371/journal.pone.0196175 (PMC5903621; doi:10.1371/journal.pone.0196175)
Supplement: S2 File — (DOCX) [file pone.0196175.s002.docx]

**Supplementary material S2:** Social discounting – recipient questionnaire
