# Supplementary material for: Putting a premium on altruism: A social discounting experiment with South African university students
Source: PLoS One. 2018 Apr 17;13(4):e0196175. doi: 10.1371/journal.pone.0196175 (PMC5903621; doi:10.1371/journal.pone.0196175)
Supplement: S3 File — (DOCX) [file pone.0196175.s003.docx]

**Supplementary material S3:** Social discounting – post-experimental subject questionnaire

**SOCIO-DEMOGRAPHIC QUESTIONNAIRE**

Please provide us with the following information – please note that all information will be kept confidential and nobody else will know what you have written:

| **1.** Age: | | | | | | | | | | | | | | | |  | | | |  | | | years | | | | |
| --- | --- | --- | --- | --- | --- | --- | --- | --- | --- | --- | --- | --- | --- | --- | --- | --- | --- | --- | --- | --- | --- | --- | --- | --- | --- | --- | --- |
|  | | | | | | | | | | | | | | | | | | | | | | | | | | | |
| **2.** Gender: | | | | | | | | | | | | | | | | Male = 1 | | | | | | | Female = 2 | | | | |
|  | | | | | | | | | | | | | | | | | | | | | | | | | | | |
| *Note*: Circle ONE option ONLY. | | | | | | | | | | | | | | | | | | | | | | | | | | | |
|  | | | | | | | | | | | | | | | | | | | | | | | | | | | |
| **3.** Race: | | African/Black = 1 | | | | Coloured = 2 | | | | | | | Asian/Indian = 3 | | | | | | | | White = 4 | | | | | | |
|  | | | | | | | | | | | | | | | | | | | | | | | | | | | |
| *Note*: Circle ONE option ONLY. | | | | | | | | | | | | | | | | | | | | | | | | | | | |
|  | | | | | | | | | | | | | | | | | | | | | | | | | | | |
| **4.** Home **l**anguage: | Afrikaans = 1 | | | English = 2 | | | | IsiNdebele = 3 | | | | IsiXhosa = 4 | | | | | | IsiZulu = 5 | | | | | | Sepedi = 6 | | | |
|  | Sesotho = 7 | | | Setswana = 8 | | | | Siswati = 9 | | | | Tshivenda = 10 | | | | | | Xitsonga = 11 | | | | | | Other = 12 | | | |
|  | | | | | | | | | | | | | | | | | | | | | | | | | | | |
| *Note*: Circle ONE option ONLY. | | | | | | | | | | | | | | | | | | | | | | | | | | | |
|  | | | | | | | | | | | | | | | | | | | | | | | | | | | |
| **5.** Faculty: | Economic and Management Sciences  = 1 | | Education  = 2 | | Health Sciences  = 3 | | | | Humanities  = 4 | | | | | | Law  = 5 | | | | Natural and Agricultural Sciences  = 6 | | | | | | Theology  = 7 | | |
|  | | | | | | | | | | | | | | | | | | | | | | | | | | | |
| *Note*: Circle ONE option ONLY. | | | | | | | | | | | | | | | | | | | | | | | | | | | |
|  | | | | | | | | | | | | | | | | | | | | | | | | | | | |
| **6.** Imagine a six-step ladder where the poorest in South Africa stand at the bottom (the first step) and the richest people in South Africa stand on the highest step (the sixth step). On which step are your household today? | | | | | | | Poorest  1 | | | 2 | | | | 3 | | 4 | | | | | | 5 | | | | | Richest  6 |
|  | | | | | | | | | | | | | | | | | | | | | | | | | | | |
| *Note*: Circle ONE option ONLY. | | | | | | | | | | | | | | | | | | | | | | | | | | | |
|  | | | | | | | | | | | | | | | | | | | | | | | | | | | |
| **7.** How will you describe your financial situation today? | | | | | | | | | | | Very broke | | | | | | | | | | | | | | | 1 | |
| (i.e. the status of your personal finances) | | | | | | | | | | | Broke | | | | | | | | | | | | | | | 2 | |
|  | | | | | | | | | | | Neither | | | | | | | | | | | | | | | 3 | |
|  | | | | | | | | | | | In good shape | | | | | | | | | | | | | | | 4 | |
| *Note*: Mark ONE option ONLY. | | | | | | | | | | | In very good shape | | | | | | | | | | | | | | | 5 | |
|  | | | | | | | | | | | | | | | | | | | | | | | | | | | |
| **8.** Have you applied to UFS for financial aid? | | | | | | | | | | | | | | | | | Yes = 1 | | | | | | No = 0 | | | | |
|  | | | | | | | | | | | | | | | | | | | | | | | | | | | |
| *Note*: Circle ONE option ONLY. | | | | | | | | | | | | | | | | | | | | | | | | | | | |
|  | | | | | | | | | | | | | | | | | | | | | | | | | | | |
| **9.** Were you awarded financial aid from UFS? | | | | | | | | | | | | | | | | | Yes = 1 | | | | | | No = 0 | | | | |
|  | | | | | | | | | | | | | | | | | | | | | | | | | | | |
| *Note*: Circle ONE option ONLY. | | | | | | | | | | | | | | | | | | | | | | | | | | | |
|  | | | | | | | | | | | | | | | | | | | | | | | | | | | |
| **10.** Have you previously participated in any experiment of this nature? | | | | | | | | | | | | | | | | | Yes = 1 | | | | | | No = 0 | | | | |
|  | | | | | | | | | | | | | | | | | | | | | | | | | | | |
| *Note*: Circle ONE option ONLY. | | | | | | | | | | | | | | | | | | | | | | | | | | | |
|  | | | | | | | | | | | | | | | | | | | | | | | | | | | |
